# Supplementary material for: Unveiling the Bioactive Potential of the Invasive Jellyfish Phyllorhiza punctata Through Integrative Transcriptomic and Proteomic Analyses
Source: Biomolecules. 2025 Aug 4;15(8):1121. doi: 10.3390/biom15081121 (PMC12383608; doi:10.3390/biom15081121)
Supplement: Supplementary file 1 [file biomolecules-15-01121-s001.zip › Suplementary Material/FileS3/PEPFOLD4/XP_065065158_12302/clusters.html]

 PEP-FOLD clusters


Download archive of all models  here   

|  |  |  |
| --- | --- | --- |
| # | file | sOPEP |
| # cluster 1 - Size: 2 |  |  |
|  | XP\_065065158\_12302-model1 | -1.97319 |
|  | XP\_065065158\_12302-model1.1 | -1.66914 |
| # cluster 2 - Size: 1 |  |  |
|  | XP\_065065158\_12302-model2 | -1.96683 |
| # cluster 3 - Size: 8 |  |  |
|  | XP\_065065158\_12302-model3 | -1.96268 |
|  | XP\_065065158\_12302-model3.1 | -1.91566 |
|  | XP\_065065158\_12302-model3.2 | -1.91566 |
|  | XP\_065065158\_12302-model3.3 | -1.85675 |
|  | XP\_065065158\_12302-model3.4 | -1.82804 |
|  | XP\_065065158\_12302-model3.5 | -1.65769 |
|  | XP\_065065158\_12302-model3.6 | -1.5974 |
|  | XP\_065065158\_12302-model3.7 | -1.38052 |
| # cluster 4 - Size: 7 |  |  |
|  | XP\_065065158\_12302-model4 | -1.45543 |
|  | XP\_065065158\_12302-model4.1 | -1.45543 |
|  | XP\_065065158\_12302-model4.2 | -1.44385 |
|  | XP\_065065158\_12302-model4.3 | -1.39968 |
|  | XP\_065065158\_12302-model4.4 | -1.39621 |
|  | XP\_065065158\_12302-model4.5 | -1.35362 |
|  | XP\_065065158\_12302-model4.6 | -1.32485 |
| # cluster 5 - Size: 3 |  |  |
|  | XP\_065065158\_12302-model5 | -1.44026 |
|  | XP\_065065158\_12302-model5.1 | -1.37718 |
|  | XP\_065065158\_12302-model5.2 | -1.37272 |
| # cluster 6 - Size: 2 |  |  |
|  | XP\_065065158\_12302-model6 | -1.43768 |
|  | XP\_065065158\_12302-model6.1 | -1.41678 |
| # cluster 7 - Size: 1 |  |  |
|  | XP\_065065158\_12302-model7 | -1.4368 |
| # cluster 8 - Size: 3 |  |  |
|  | XP\_065065158\_12302-model8 | -1.42352 |
|  | XP\_065065158\_12302-model8.1 | -1.38713 |
|  | XP\_065065158\_12302-model8.2 | -1.38372 |
| # cluster 9 - Size: 2 |  |  |
|  | XP\_065065158\_12302-model9 | -1.41555 |
|  | XP\_065065158\_12302-model9.1 | -1.37549 |
| # cluster 10 - Size: 9 |  |  |
|  | XP\_065065158\_12302-model10 | -1.41304 |
|  | XP\_065065158\_12302-model10.1 | -1.40984 |
|  | XP\_065065158\_12302-model10.2 | -1.40388 |
|  | XP\_065065158\_12302-model10.3 | -1.39495 |
|  | XP\_065065158\_12302-model10.4 | -1.3764 |
|  | XP\_065065158\_12302-model10.5 | -1.37565 |
|  | XP\_065065158\_12302-model10.6 | -1.36661 |
|  | XP\_065065158\_12302-model10.7 | -1.36413 |
|  | XP\_065065158\_12302-model10.8 | -1.33682 |
| # cluster 11 - Size: 4 |  |  |
|  | XP\_065065158\_12302-model11 | -1.40578 |
|  | XP\_065065158\_12302-model11.1 | -1.39822 |
|  | XP\_065065158\_12302-model11.2 | -1.38127 |
|  | XP\_065065158\_12302-model11.3 | -1.33625 |
| # cluster 12 - Size: 5 |  |  |
|  | XP\_065065158\_12302-model12 | -1.39869 |
|  | XP\_065065158\_12302-model12.1 | -1.38706 |
|  | XP\_065065158\_12302-model12.2 | -1.37586 |
|  | XP\_065065158\_12302-model12.3 | -1.36346 |
|  | XP\_065065158\_12302-model12.4 | -1.35853 |
| # cluster 13 - Size: 3 |  |  |
|  | XP\_065065158\_12302-model13 | -1.39636 |
|  | XP\_065065158\_12302-model13.1 | -1.37615 |
|  | XP\_065065158\_12302-model13.2 | -1.33769 |
| # cluster 14 - Size: 12 |  |  |
|  | XP\_065065158\_12302-model14 | -1.39411 |
|  | XP\_065065158\_12302-model14.1 | -1.39411 |
|  | XP\_065065158\_12302-model14.2 | -1.39411 |
|  | XP\_065065158\_12302-model14.3 | -1.39411 |
|  | XP\_065065158\_12302-model14.4 | -1.38023 |
|  | XP\_065065158\_12302-model14.5 | -1.38023 |
|  | XP\_065065158\_12302-model14.6 | -1.36924 |
|  | XP\_065065158\_12302-model14.7 | -1.36924 |
|  | XP\_065065158\_12302-model14.8 | -1.3629 |
|  | XP\_065065158\_12302-model14.9 | -1.3629 |
|  | XP\_065065158\_12302-model14.10 | -1.3629 |
|  | XP\_065065158\_12302-model14.11 | -1.3561 |
| # cluster 15 - Size: 3 |  |  |
|  | XP\_065065158\_12302-model15 | -1.39162 |
|  | XP\_065065158\_12302-model15.1 | -1.37329 |
|  | XP\_065065158\_12302-model15.2 | -1.36649 |
| # cluster 16 - Size: 1 |  |  |
|  | XP\_065065158\_12302-model16 | -1.38256 |
| # cluster 17 - Size: 10 |  |  |
|  | XP\_065065158\_12302-model17 | -1.3753 |
|  | XP\_065065158\_12302-model17.1 | -1.3753 |
|  | XP\_065065158\_12302-model17.2 | -1.3753 |
|  | XP\_065065158\_12302-model17.3 | -1.36738 |
|  | XP\_065065158\_12302-model17.4 | -1.36738 |
|  | XP\_065065158\_12302-model17.5 | -1.36738 |
|  | XP\_065065158\_12302-model17.6 | -1.36738 |
|  | XP\_065065158\_12302-model17.7 | -1.36738 |
|  | XP\_065065158\_12302-model17.8 | -1.36738 |
|  | XP\_065065158\_12302-model17.9 | -1.32061 |
| # cluster 18 - Size: 6 |  |  |
|  | XP\_065065158\_12302-model18 | -1.37025 |
|  | XP\_065065158\_12302-model18.1 | -1.37025 |
|  | XP\_065065158\_12302-model18.2 | -1.37025 |
|  | XP\_065065158\_12302-model18.3 | -1.35849 |
|  | XP\_065065158\_12302-model18.4 | -1.3461 |
|  | XP\_065065158\_12302-model18.5 | -1.31511 |
| # cluster 19 - Size: 10 |  |  |
|  | XP\_065065158\_12302-model19 | -1.3697 |
|  | XP\_065065158\_12302-model19.1 | -1.3697 |
|  | XP\_065065158\_12302-model19.2 | -1.36399 |
|  | XP\_065065158\_12302-model19.3 | -1.36399 |
|  | XP\_065065158\_12302-model19.4 | -1.36399 |
|  | XP\_065065158\_12302-model19.5 | -1.36165 |
|  | XP\_065065158\_12302-model19.6 | -1.35299 |
|  | XP\_065065158\_12302-model19.7 | -1.3528 |
|  | XP\_065065158\_12302-model19.8 | -1.35156 |
|  | XP\_065065158\_12302-model19.9 | -1.34051 |
| # cluster 20 - Size: 2 |  |  |
|  | XP\_065065158\_12302-model20 | -1.35139 |
|  | XP\_065065158\_12302-model20.1 | -1.33255 |
| # cluster 21 - Size: 3 |  |  |
|  | XP\_065065158\_12302-model21 | -1.34699 |
|  | XP\_065065158\_12302-model21.1 | -1.343 |
|  | XP\_065065158\_12302-model21.2 | -1.32832 |
| # cluster 22 - Size: 2 |  |  |
|  | XP\_065065158\_12302-model22 | -1.33711 |
|  | XP\_065065158\_12302-model22.1 | -1.33537 |
| # cluster 23 - Size: 1 |  |  |
|  | XP\_065065158\_12302-model23 | -1.32934 |
|  |  |  |
